# Prognostic impact of blood group O in MGMT-methylated glioblastoma patients receiving radiochemotherapy with temozolomide and lomustine

**DOI:** 10.1007/s11060-026-05813-y

**Published:** 2026-09-24

**Authors:** Julia Scheuble, Thomas Zeyen, Matthias Schneider, Anna-Laura Potthoff, Niklas Schäfer, Lea Lex, Lea L. Friker, Alexander Radbruch, Julian P. Layer, Eleni Gkika, Hartmut Vatter, Ulrich Herrlinger, Johannes Weller

**Affiliations:** 1 Department of Neuro-Oncology, University Hospital Bonn, University of Bonn, Venusberg Campus 1, 53127 Bonn, Germany; 2 Brain Tumor Translational Research Group, University Hospital Bonn, University of Bonn, Venusberg Campus 1, 53127 Bonn, Germany; 3 Department of Neurosurgery, University Hospital Bonn, University of Bonn, Bonn, Germany; 4 Institute for Neuropathology, University Hospital Bonn, University of Bonn, Bonn, Germany; 5 Institute of Experimental Oncology, University Hospital Bonn, University of Bonn, Bonn, Germany; 6 Department of Neuroradiology, University Hospital Bonn, University of Bonn, Bonn, Germany; 7 Clinical Neuroimaging Group, German Center for Neurodegenerative Diseases, University Bonn, Bonn, Germany; 8 Department of Radiation Therapy and Radiooncology, University Hospital Bonn, University of Bonn, Bonn, Germany; 9 Department of Vascular Neurology, University Hospital Bonn, University of Bonn, Venusberg Campus 1, 53127 Bonn, Germany; 10 Department of Neuroimmunology, University Hospital Bonn, University of Bonn, Venusberg Campus 1, 53127 Bonn, Germany

**Keywords:** Glioblastoma, MGMT promoter methylation, ABO blood group, Lomustine, Temozolomide

## Abstract

**Purpose:**

Blood group O has recently been associated with improved survival in patients with MGMT-methylated glioblastoma. However, whether this association persists in patients treated with intensified first-line radiochemotherapy according to the CeTeG/NOA-09 regimen remains unclear. We aimed to validate the prognostic impact of ABO blood group in an independent cohort of patients with MGMT-methylated, IDH-wildtype glioblastoma uniformly treated with CCNU/temozolomide (CCNU/TMZ).

**Methods:**

We performed a retrospective single-center cohort study including adult patients with newly diagnosed MGMT-methylated, IDH-wildtype glioblastoma treated according to the CeTeG/NOA-09 protocol between 2008 and 2023. Progression-free survival (PFS) and overall survival (OS) were analyzed using Kaplan–Meier estimates, log-rank tests, and multivariable Cox proportional hazards models adjusted for age, extent of resection, and Karnofsky Performance Status.

**Results:**

Sixty-one patients were included, of whom 24 (39.3%) had blood group O and 37 (60.7%) had non-O blood groups. Blood group O was associated with longer OS (median 42.1 vs. 23.7 months; log-rank *p* = 0.04) and a trend toward longer PFS (median 21.8 vs. 17.5 months; log-rank *p* = 0.10). After multivariable adjustment, non-O blood groups remained independently associated with shorter PFS (HR 1.86, 95% CI 1.04–3.34; *p* = 0.037) and OS (HR 2.29, 95% CI 1.16–4.52; *p* = 0.017).

**Conclusion:**

Blood group O remained associated with improved progression-free and overall survival in patients with MGMT-methylated, IDH-wildtype glioblastoma treated according to the CeTeG/NOA-09 regimen. These findings support an association between blood group O and improved survival outcomes and warrant further investigation of its biological and clinical relevance in glioblastoma.

## Introduction

Glioblastoma remains associated with poor survival despite multimodal treatment and established prognostic factors such as age, performance status, extent of resection, and MGMT promoter methylation [1, 2]. Nevertheless, clinical outcomes remain highly heterogeneous, highlighting the need for additional prognostic biomarkers. Beyond its established role in transfusion medicine, the ABO blood group system has increasingly emerged as a potential modifier of cancer biology. Associations between ABO blood group and cancer risk or survival have been reported across several solid malignancies, although the underlying mechanisms remain incompletely understood [3]. However, whether these mechanisms contribute to glioblastoma biology remains unknown, and the prognostic relevance of ABO blood group in glioblastoma has remained controversial [4]. Furthermore, most previous studies were conducted before implementation of the WHO 2021 integrated molecular classification of central nervous system tumors [5], limiting their applicability to contemporary molecularly defined glioblastoma cohorts.

Recently, Wiewrodt et al. reported improved survival in patients with blood group O compared with those with non-O blood groups among patients with MGMT-methylated, but not MGMT-unmethylated, glioblastoma [6]. Notably, this association was observed predominantly in patients receiving standard temozolomide-based radiochemotherapy. Among 223 patients with MGMT-methylated tumors, only 36 (16.1%) received intensified first-line treatment with CCNU/TMZ according to the CeTeG/NOA-09 protocol [7], limiting the statistical power to assess this subgroup. Consequently, whether the prognostic association of blood group O persists under intensified first-line alkylating chemotherapy remains unknown. To our knowledge, this study provides the first independent validation of this association in a homogeneous cohort of MGMT-methylated glioblastoma patients uniformly treated according to the CeTeG/NOA-09 regimen. Independent validation is an essential step before novel prognostic markers can be considered robust and potentially relevant for future clinical risk stratification.

## Methods

### Study design

We conducted a retrospective single-center cohort study including adult patients with newly diagnosed MGMT-methylated, IDH-wildtype glioblastoma treated at the University Hospital Bonn between 2008 and 2023. The present study represents a protocol-defined, retrospective cohort rather than an unselected series of all patients with glioblastoma treated at our institution during the study period. Patients were identified from institutional treatment-protocol cohorts and screened for eligibility. Eligible patients were required to have an MGMT-methylated, IDH-wildtype glioblastoma, to have received first-line radiochemotherapy according to the CeTeG/NOA-09 regimen, and to have a documented ABO blood group available from routine preoperative blood typing. Although the study period extended from 2008 to 2023, the majority of eligible patients were treated from 2018 onwards, reflecting the increasing routine availability and systematic documentation of molecular tumor characterization during this period. MGMT promoter methylation status was determined as part of the diagnostic work-up at initial diagnosis. For patients enrolled in the CeTeG/NOA-09 trial, MGMT promoter methylation was determined by central testing using methylation-specific real-time PCR as previously described [7], with an MGMT/ACTB ratio > 2.19 defining promoter methylation. Outside the clinical trial, MGMT promoter methylation was routinely assessed at our center using quantitative pyrosequencing as previously described by Mikeska et al. [8], with a methylation level of ≥ 9% defining MGMT promoter-methylated tumors. No de novo MGMT testing of archived tumor tissue was performed for the present study. Clinical, radiological, and molecular data were extracted from the electronic health records. The study was approved by the Ethics Committee of the University Hospital Bonn (reference no. 09/22).

### Outcome assessment

Progression-free survival (PFS) and overall survival (OS) were calculated from the date of histological diagnosis to the respective event or censoring. Tumor progression was assessed according to the Response Assessment in Neuro-Oncology (RANO) criteria [9]. Patients without an event were censored at the date of last clinical follow-up.

### Treatment protocol

All patients received first-line radiochemotherapy according to the CeTeG/NOA-09 protocol. Treatment consisted of radiotherapy combined with lomustine and temozolomide as previously described by Herrlinger et al. [7] Dose modifications and supportive care were performed according to institutional standards. Treatment at disease progression was individualized at the discretion of the treating physicians and included re-resection, re-irradiation, and systemic salvage therapies. Salvage treatment modalities were retrospectively extracted from the medical records and compared between patients with blood group O and non-O blood groups.

### Statistical analysis

Continuous variables are presented as median (interquartile range) or mean (standard deviation), as appropriate, whereas categorical variables are reported as frequencies and percentages. Categorical variables, including salvage treatment modalities, were compared using Fisher’s exact test. Survival was estimated using the Kaplan–Meier method and compared using the log-rank test. Patients with missing covariate data were excluded from multivariable analyses. Multivariable Cox proportional hazards models were fitted to assess the independent prognostic impact of blood group O after adjustment for age (< 65 vs. ≥65 years), extent of resection (gross total resection [GTR] vs. non-GTR), and Karnofsky Performance Status (KPS) (< 80% vs. 80–100%). Missing data were not imputed, and complete-case analysis was performed for the multivariable Cox proportional hazards models. Median follow-up was estimated using the reverse Kaplan–Meier method for the overall cohort and separately according to blood group. Hazard ratios (HRs) are reported with 95% confidence intervals (CIs). A two-sided p-value < 0.05 was considered statistically significant. Statistical analyses were performed using R version 4.2.1.

## Results

### Patient characteristics

A total of sixty-one patients met the predefined eligibility criteria for the present analysis. The relatively small study cohort reflects the restriction to patients with MGMT-methylated, IDH-wildtype glioblastoma who received first-line CCNU/TMZ and had documented ABO blood group information. Although the overall study period extended from 2008 to 2023, the cohort was concentrated in the later years, with 48 of 61 patients with available surgery dates (78.7%) undergoing surgery between 2018 and 2023. Of these, 24 (39.3%) had blood group O and 37 (60.7%) had non-O blood groups. Baseline characteristics were well balanced between the two groups (Table 1), with no significant differences in age, sex, KPS, or extent of resection. The proportion of patients with blood group O (39.3%) was comparable to the reported prevalence among German blood donors (39.2%) [10]. 

**Table 1:** Baseline characteristics of patients with MGMT-methylated IDH-wildtype glioblastoma treated according to the CeTeG/NOA-09 regimen (*n* = 61)

Variable	Blood group O cohort (*n* = 24)	Non-blood group O cohort (*n* = 37)	*p*-value
Age at diagnosis, median (IQR)	57.0 years (53.2–61.8)	62.0 years (55.8–66.0)	0.172
Female sex, n (%)	10 (41.7)	15 (40.5)	1.0
KPS, median (IQR)	90% (80–90)	90% (80–90)	0.367
Gross total resection, n (%)	12 (50.0)	21 (56.8)	0.793

Abbreviations: IQR, interquartile range; KPS, Karnofsky Performance Status

### Survival outcomes

**Fig. 1:**
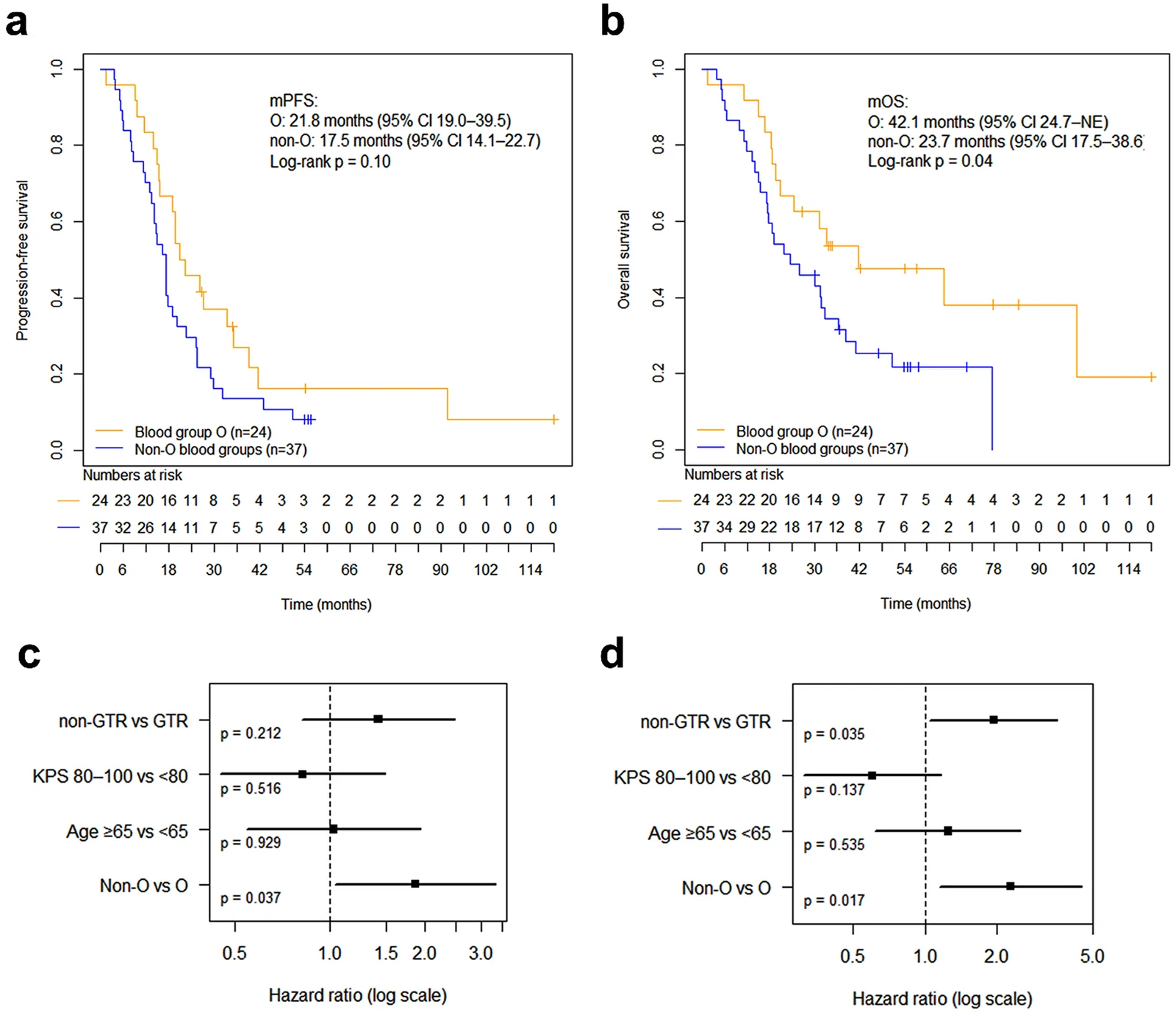
Kaplan-Meier curves for overall survival (OS) (**a**) and progression-free survival (PFS) (**b**) in MGMT-methylated glioblastoma patients receiving first-line CCNU/TMZ in addition to radiotherapy with blood group O (orange) and non-O blood groups (blue). Ticks represent censoring of patients. Forest plots depict multivariable Cox proportional hazards analyses for PFS (**c**) and OS (**d**), adjusted for age, Karnofsky Performance Status, and extent of resection. Hazard ratios are shown with corresponding 95% confidence intervals; p-values are displayed beneath each covariate. Abbreviations: O: Blood Group O; non-O: non-Blood Group O; NE: not estimable

Kaplan–Meier analysis demonstrated a trend toward longer progression-free survival and significantly longer overall survival in patients with blood group O than in those with non-O blood groups (Fig. 1a-b). Median follow-up estimated by the reverse Kaplan–Meier method was 56.1 months in the overall cohort. Median follow-up was comparable between patients with blood group O (57.9 months, 95% CI 35.1–NE) and non-O blood groups (55.3 months, 95% CI 54.3–NE). Median PFS was 21.8 months (95% CI 19.0–39.5) in patients with blood group O compared with 17.5 months (95% CI 14.1–22.7) in patients with non-O blood groups (log-rank *p* = 0.10). Median OS was 42.1 months (95% CI 24.7–not estimable) in patients with blood group O compared with 23.7 months (95% CI 17.5–38.6) in patients with non-O blood groups (log-rank *p* = 0.04). Overall, salvage treatment was administered to 10/19 (52.6%) patients with blood group O and 13/32 (40.6%) patients with non-O blood groups. Treatment modalities included re-resection, re-irradiation, and systemic therapy. Re-resection was performed in 1/19 (5.3%) and 1/32 (3.1%) patients, respectively (*p* = 1.00), while re-irradiation was performed in 3/19 (15.8%) and 9/32 (28.1%) patients (*p* = 0.50). Systemic treatments included temozolomide rechallenge, either alone or in combination with re-irradiation (*n* = 7 vs. *n* = 8), bevacizumab (*n* = 2 vs. *n* = 1), and regorafenib (*n* = 1 vs. *n* = 0) in patients with blood group O versus non-O blood groups, respectively. No statistically significant differences in the major salvage treatment modalities were observed between the two groups.

### Multivariable analysis

One patient with missing KPS data was excluded from the multivariable Cox analyses, resulting in 60 patients being included in both models. The OS model included 43 events (10.8 events per model parameter), while the PFS model included 54 events (13.5 events per model parameter). There was no evidence of violation of the proportional hazards assumption for either model based on Schoenfeld residuals (global test: OS, *p* = 0.72; PFS, *p* = 0.92). No other data required for the survival analyses were missing. After adjustment for age, extent of resection, and KPS, blood group remained associated with progression-free and overall survival. Compared with blood group O, non-O blood groups were associated with shorter PFS (HR 1.86, 95% CI 1.04–3.34; *p* = 0.037) and OS (HR 2.29, 95% CI 1.16–4.52; *p* = 0.017) (Fig. 1c-d). In sensitivity analyses sequentially omitting age, KPS, or extent of resection from the multivariable models, the association between non-O blood group and OS remained consistent (HR range 2.02–2.42; *p* = 0.011–0.039). For PFS, effect estimates were likewise similar across reduced models (HR range 1.72–1.90), although statistical significance was not retained after omission of extent of resection (HR 1.72, 95% CI 0.98–3.05; *p* = 0.060).

## Discussion

Our findings closely align with those reported by Wiewrodt et al. [6] and provide an independent validation of this association in patients with MGMT-methylated glioblastoma treated with combined CCNU/TMZ radiochemotherapy according to the CeTeG/NOA-09 protocol [7]. In our cohort, blood group O was associated with prolonged progression-free and overall survival, consistent with the previously reported association in this molecularly defined subgroup. While the unadjusted comparison of PFS did not reach statistical significance (log-rank *p* = 0.10), an association was observed in the adjusted Cox model. Given the limited cohort size and potential for residual confounding, this finding should be interpreted cautiously and requires confirmation in larger independent cohorts.

Replication of newly identified prognostic associations in independent cohorts represents an important prerequisite before such markers can be considered reproducible. In this context, the present study extends the observations by Wiewrodt et al. to a clinically distinct cohort uniformly treated according to the CeTeG/NOA-09 regimen, thereby minimizing treatment-related heterogeneity.

The biological basis of the observed association remains uncertain. Several biological mechanisms have been proposed, including effects of ABO glycosyltransferase activity on glycosylation, cell-cell adhesion, cellular mobility, and susceptibility to apoptosis, which may ultimately influence tumor progression or treatment response [6]. Experimental studies in non-glioma tumor models have linked glycosyltransferase activity to increased cellular mobility and resistance to apoptosis [11, 12], providing a potential biological rationale for an interaction between ABO-dependent glycosylation and treatment-induced cell death. In MGMT promoter-methylated glioblastoma, in which reduced MGMT-mediated DNA repair increases susceptibility to alkylating agents, such mechanisms could theoretically modify the cellular response to temozolomide or lomustine. However, there is currently no experimental evidence demonstrating a direct interaction between ABO glycosyltransferase activity, MGMT promoter methylation, and sensitivity to alkylating chemotherapy in glioblastoma. Thus, the observed association cannot currently be attributed to a treatment-specific effect and may alternatively reflect differences in intrinsic tumor biology or the tumor microenvironment.

The absence of independent effects of age and KPS in the multivariable analyses most likely reflects the restricted variability and clinical selection inherent to patients eligible for CCNU/TMZ treatment rather than a lack of prognostic relevance of these established factors.

This study has several limitations, including its retrospective single-center design and limited sample size. At the same time, the present study has several strengths that should be acknowledged. The study comprises a molecularly homogeneous cohort of patients who received intensified first-line CCNU/TMZ therapy, thereby minimizing treatment-related heterogeneity. Furthermore, adjustment for established prognostic variables supports the robustness of the observed association between blood group O and survival. Despite multivariable adjustment, residual confounding cannot be excluded given the retrospective design and limited sample size. The restriction to patients considered suitable for intensified CCNU/TMZ treatment resulted in a clinically selected population with relatively favorable performance status. Consequently, our findings may not be generalizable to unselected patients with glioblastoma, particularly clinically frail patients who would not be considered candidates for CCNU/TMZ combination therapy. In addition, the 15-year study period may have introduced historical confounding due to evolving surgical techniques, neuroimaging practices, and treatment strategies at recurrence. However, the cohort was substantially concentrated in the later years of the study period, which may have limited the extent of temporal heterogeneity. Validation in larger, preferably multicenter cohorts will therefore be important to assess the robustness and generalizability of the observed association.

In conclusion, blood group O was associated with improved survival outcomes in this retrospective cohort of patients with MGMT-methylated IDH-wildtype glioblastoma treated with CCNU/TMZ. Given the limited sample size, retrospective design, and potential for residual confounding, these findings should be considered hypothesis-generating and require validation in larger independent cohorts. Future translational investigations are warranted to elucidate the biological mechanisms underlying this association.

## Data Availability

No datasets were generated or analysed during the current study.
